# Heterogeneity of thymic output in the elderly and its association with sex and smoking

**DOI:** 10.1172/jci.insight.189008

**Published:** 2025-07-01

**Authors:** Balraj Sandhar, Vishal Vyas, Daniel Harding, Roberta Ragazzini, Paola Bonfanti, Federica M. Marelli-Berg, Christopher G. Bell, Benny M. Chain, M. Paula Longhi

**Affiliations:** 1 William Harvey Research Institute, Barts and The London School of Medicine and Dentistry, Queen Mary University of London, London, United Kingdom.; 2 Department of Cardiology, Barts Heart Centre, St. Bartholomew’s Hospital, London, United Kingdom.; 3 Epithelial Stem Cell Biology & Regenerative Medicine Laboratory, The Francis Crick Institute, London, United Kingdom.; 4 Institute of Immunity & Transplantation, Division of Infection & Immunity, UCL, Pears Building, Rosslyn Hill, London, United Kingdom.; 5 Division of Infection and Immunity, University College London, London, United Kingdom.

**Keywords:** Cardiology, Immunology, Adaptive immunity, Adipose tissue

## Abstract

**BACKGROUND:**

Thymic involution with age leads to reduced T cell output and impaired adaptive immunity. However, the extent to which thymic activity persists later in life and how this contributes to immunological aging remains unclear. This study aimed to assess the presence and function of thymic tissue in older adults and identify factors influencing residual thymopoiesis.

**METHODS:**

Patients aged 50 or older undergoing cardiothoracic surgery were recruited. Thymic structures within mediastinal adipose tissue were evaluated using histology, immunofluorescence, flow cytometry, T cell receptor (TCR) sequencing, and RNA sequencing. Recent thymic emigrants (RTEs) were quantified in peripheral blood and correlated with transcriptomic, epigenetic, and TCR repertoire data. Primary outcomes included thymic tissue identification, RTE frequency, and immune correlates.

**RESULTS:**

Functional thymic tissue was identified in mediastinal adipose tissue of older individuals. The frequency of CD31^+^CD4^+^ T cells (RTEs) positively correlated with the presence of thymic tissue. Thymic output showed substantial heterogeneity and was influenced by sex and smoking history. Thymic activity was associated with increased TCR repertoire diversity, improved immune protection against infections, and reduced epigenetic aging. Detailed profiling uncovered functional and phenotypic heterogeneity within naive CD4^+^ T cell subsets shaped by thymic activity.

**CONCLUSION:**

This study demonstrates that thymic function can persist into later life and is modulated by factors such as sex and smoking. These findings suggest that thymic activity during aging is heterogeneous and influenced by more than chronological age alone, with potential implications for immune competence in older adults.

## Abstract 2

This work underscores smoking and sex differences as significant contributors to thymopoietic activity in the elderly impacting T cell diversity and mortality risk.

## Graphical abstract

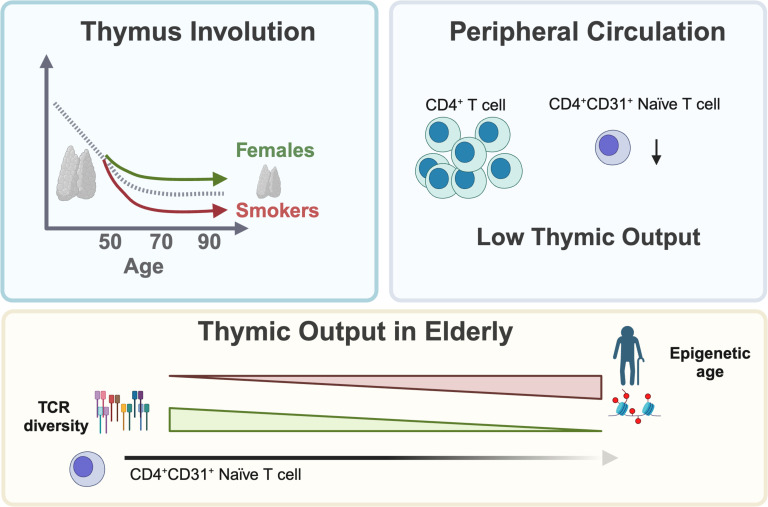

## Introduction

As a primary lymphoid organ, the thymus has a significant role in guiding the differentiation of lymphocyte precursors and facilitating their positive and negative selection processes (1–3). Therefore, as the fundamental site for T cell development, the thymus establishes adaptive immunity and central tolerance by generating mature immunocompetent T cells. Over an individual’s lifespan, the thymus undergoes involution, a complex process involving structural changes and disorganization to cortical and medullary compartments accompanied by increased fatty deposition (4, 5). Despite progressive fatty replacement of the thymus with age (6, 7), residual thymic tissue can be detected later in life. The degree of thymic activity in older age is less clear, with some reports showing minimal function (8, 9), while others describe variable residual thymus tissue after 50 years of age ranging from 16% to 50% (10–13). The removal of the thymus in adults has significant implications for the immune system’s functionality (10). However, how gradual thymus regression with age affects health in older adults is less known. In humans, as thymic activity diminishes, the replenishment of naive T cell populations in the periphery largely depends on homeostatic proliferation, a process that maintains T cell numbers by cell division (12, 14). This dynamic balance between thymic output and peripheral homeostasis becomes increasingly crucial in the context of immune function, as the gradual decline in naive T cell numbers is accompanied by oligoclonal expansion of effector/memory T cell subsets (15–17). Consequently, the overall robustness of adaptive immune responses can be compromised, posing challenges in combating novel pathogens and antigens (18).

While age-related pathways have been recognized as primary drivers of thymic involution, recent investigations have unveiled additional factors contributing to accelerated thymic regression. Studies have correlated markers for metabolic disease, stressors, and sex differences with heightened rates of thymic involution, indicating that the processes influencing thymopoiesis are multifaceted (19–25). Despite this growing body of research, the role of altered thymic output in older individuals and the underlying factors driving thymus regression later in life remain poorly understood.

In this study, we sought to characterize thymic function in aged individuals when the rate of thymus involution decreases (9, 26). By analyzing distinct T cell populations in blood and correlating these with thymic activity from within mediastinal adipose tissue (AT), we found highly heterogeneous thymus output in individuals over 50 years of age modulated by sex and smoking. Additionally, we investigated how thymus involution contributes to the immune dysregulation described in old age, finding decreased T cell receptor (TCR) diversity, increased susceptibility to intrahospital respiratory infections, and increased mortality risk in those individuals with low thymic function.

## Results

### Functional thymic structures are embedded in the mediastinal AT.

**Figure 1:**
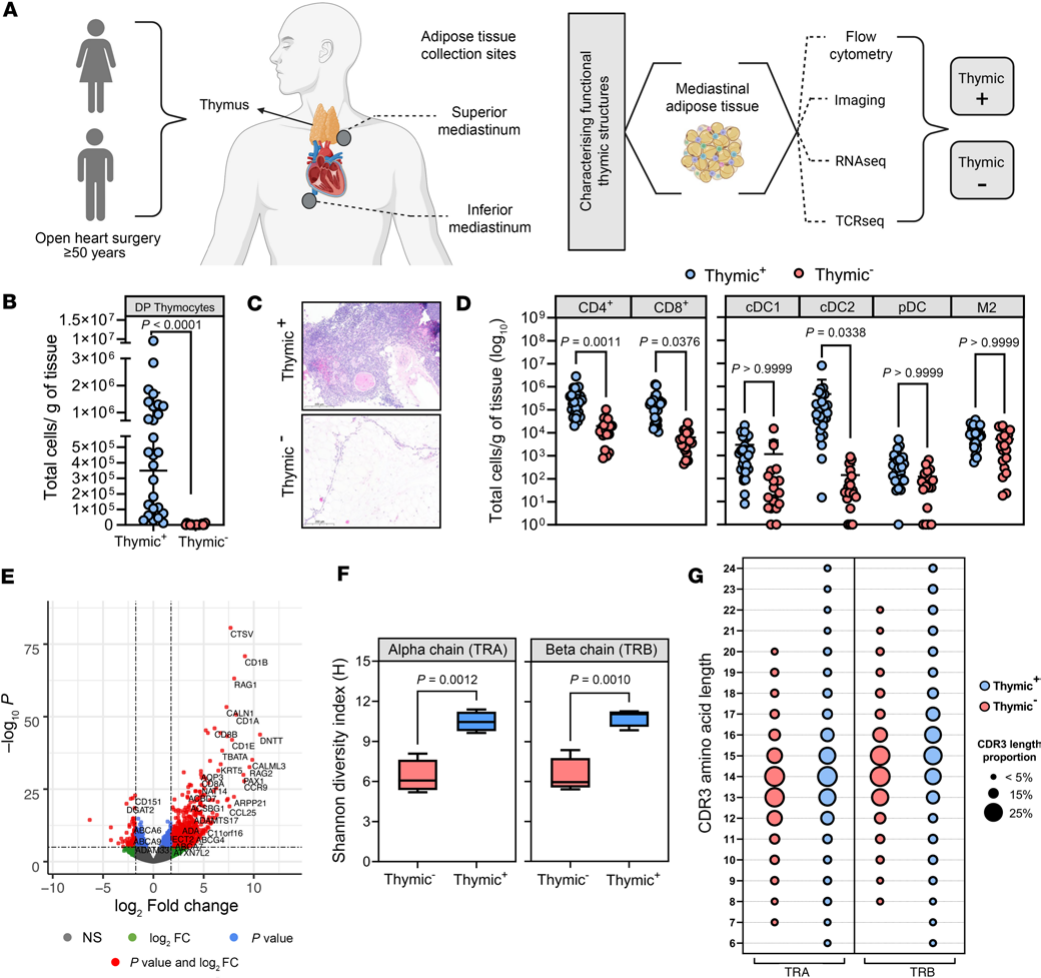
Characterizing immune cellular landscape and function of thymic structures in aged patients. (**A**) Schematic illustrating project workflow. (**B**) Identification of thymocyte populations via flow cytometry in thymic^+^ (*n* = 27) and thymic^–^ (*n* = 19) samples. Statistical significance was evaluated by 2-tailed Mann-Whitney *U* test. (**C**) Representative thymic structures observed through H&E staining. Scale bars: 200 μm. (**D**) Immunophenotyping single-positive CD4^+^ and CD8^+^ T cells, conventional dendritic cells (cDC1 and cDC2), plasmacytoid dendritic cells (pDCs), and M2 macrophages (M2) in thymic^+^ and thymic^–^ samples. Statistical significance was evaluated by 2-way ANOVA and Šidák’s test for multiple comparisons. Data in **B** and **D** presented as mean ± SD. (**E**) Volcano plot depicting the average log-fold gene expression changes and Benjamini-Hochberg–corrected *P* values for pairwise comparisons between thymic^–^ control adipose tissue (*n* = 4) and thymic^+^ tissues (*n* = 3). (**F**) Shannon diversity index for TCR α (TRA) and β (TRB) chains between thymic^+^ and thymic^–^ samples. Significance evaluated by unpaired, 2-tailed Student’s *t* test. Boxes represent the 25^th^ and 75^th^ percentiles, lines inside the boxes represent medians, whiskers represent the upper and lower adjacent values. (**G**) CDR3 length for TRA and TRB chains (thymic^+^ *n* = 4; thymic^–^ *n* = 4).

To effectively evaluate thymic output in aged individuals, we first sought to characterized functional thymic tissue obtained from patients (≥50 years) undergoing common cardiac surgical procedures, where these tissues could be ethically accessed. ATs were collected during surgery from distinct locations within the chest cavity, alongside preoperative fasting blood samples. Specifically, mediastinal ATs from superior and inferior regions were acquired for phenotyping (Figure 1A). These locations were selected because they include sites of the native thymus, involuted thymic remnants, and ectopic thymic deposits (27). The presence of thymic tissue in AT samples was first assessed by flow cytometry, evidenced by the identification of CD8^+^CD4^+^ (double-positive, DP) T cells (Figure 1B). Thymic^+^ tissues were considered as those having a significant proportion of DP T cells (20%–80% of CD45^+^ cells), with thymic^–^ having less than 5% of DP cells from all AT samples tested (Figure 1B). DP T cells in the superior mediastinal AT could be identified in 48% of patients. From those, a second focus of DP T cells in the lower mediastinal AT was identified in 35% of patients. In 11% of patients, the primary DP focus was present in the lower mediastinal AT. Clinical characteristics of patients are summarized in Supplemental Table 1 (supplemental material available online with this article; https://doi.org/10.1172/jci.insight.189008DS1), showing an increased prevalence of females in the thymic^+^ group. The presence of thymic tissue was confirmed by histological examination (Figure 1C). In addition, through different gating strategies (Supplemental Figure 1, A and B), the number of mature T cells, conventional dendritic cells (cDC1 and cDC2), plasmacytoid dendritic cells (pDCs), and M2 macrophages were assessed, with samples with high levels of DP T cells showing increased CD4^+^ T cells, CD8^+^ T cells, and cDC2’s per gram of tissue (Figure 1D).

To further validate the thymopoietic properties of thymic^+^ tissue, bulk RNA-seq and TCR-seq analyses were performed. Eight hundred thirteen differentially expressed genes (DEGs) were identified between thymic^–^ (*n* = 4) and thymic^+^ (*n* = 3) AT samples (Figure 1E and Supplemental Table 2). Genes associated with development and maintenance of the thymus stromal microenvironment (e.g., *CTSV*, *FOXN1*, *PSMB11*, *TBATA*, *KRT17*, *PAX1*) and VDJ recombination (e.g., *RAG1*, *RAG2*, *DNTT*) were upregulated in thymic^+^ samples, whereas genes related to adipogenesis (e.g., *ADIPOQ*, *PPARG*, *FABP4*) were downregulated. TCR-seq analysis further confirmed the functional capacity of thymic^+^ tissue, mostly through DP T cell populations, in supporting thymocyte differentiation and maturation. This was evidenced by enhanced TCR diversity (Figure 1F), and variable complementarity-determining region 3 (CDR3) length usage indicative of immature thymocytes (Figure 1G) (28). While these data support active thymopoiesis in thymic^+^ tissue, caution in interpretation is warranted due to the small sample size.

Together, these findings demonstrate that, despite a decrease in thymic mass and cellularity with age, functional thymic structures could be detected embedded in AT with the capacity to facilitate T cell development.

### Thymic function is influenced by sex and smoking status in aged individuals.

**Figure 2:**
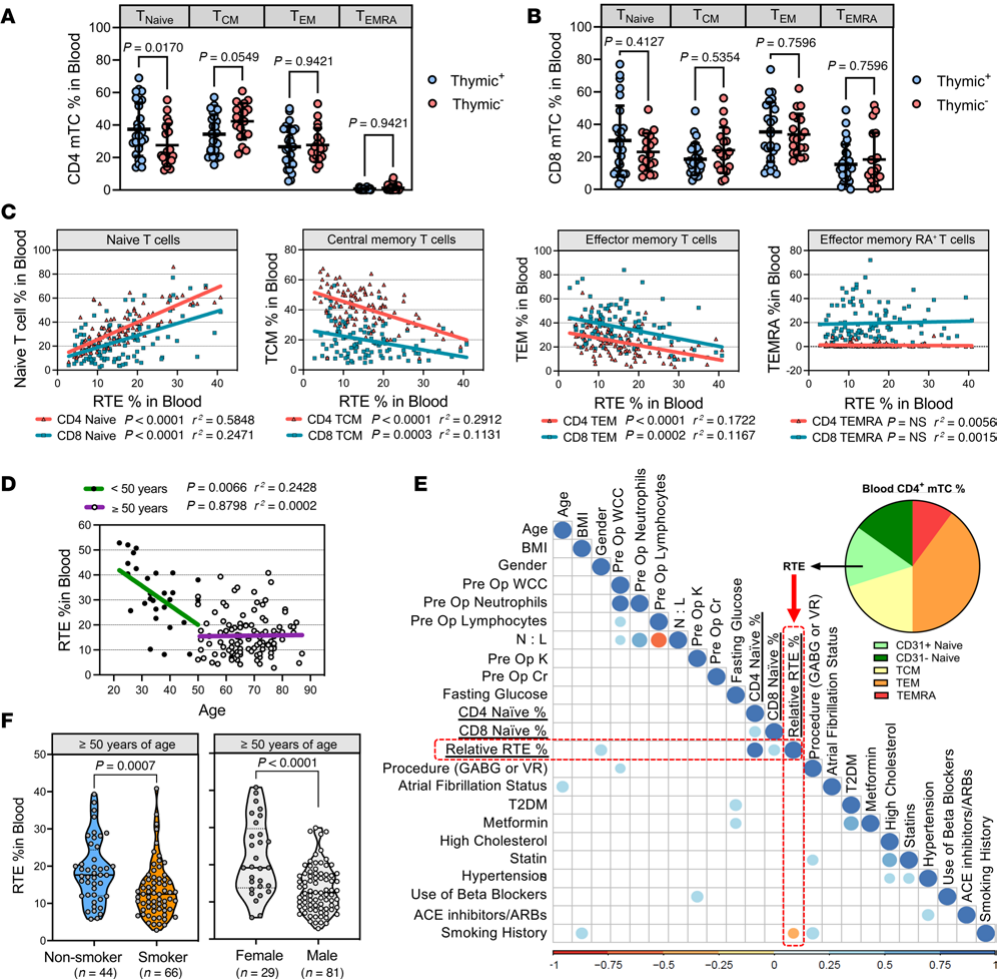
Thymic output correlates to peripheral T cell subpopulation and associates with sex and smoking in older patients. (**A**) Proportion of CD4^+^ and (**B**) CD8^+^ naive, TCM, TEM, and TEMRA cells in the blood of patients classified as either thymic^+^ or thymic^–^. Data presented as mean ± SD. Statistical significance evaluated by 2-way ANOVA with Šídák’s multiple-comparison test. (**C**) Linear regression analysis comparing RTE% in blood to peripheral T cell pools. (**D**) Correlative linear regression analysis between chronological age and RTE% in blood for individuals aged <50 years (young; green) (*n* = 35) and ≥50 years (old; purple) (*n* = 110). (**E**) Correlation matrix comparing clinical characteristics to thymic output (relative RTE%) and naive T cell percentage in the blood of patients ≥50 years. Spearman’s coefficients with Bonferroni’s correction *P* value cutoff <0.002. Significant correlation coefficients are shown as either blue (positive correlation) or red (negative correlation) dots. (**F**) Graph illustrating RTE% in blood between smokers and nonsmokers and males and females. Truncated violin plots show medians and interquartile ranges, and significance was evaluated by 2-tailed Mann-Whitney *U* test. The *r*^2^ values in **D** and **E** were calculated using Pearson’s correlation coefficients and significance by 2-sided *P* value analysis.

To explore the impact of thymic function on peripheral T cell subsets, the proportion of CD4^+^ and CD8^+^ T cells and their respective phenotypes were assessed in the blood of patients characterized as either thymic^+^ or thymic^–^. Thymic^+^ patients were shown to have a notable increase in peripheral naive CD4^+^ T cells compared with thymic^–^ individuals (Figure 2A). No significant differences were observed in the CD8^+^ T cell compartment (Figure 2B). We then investigated whether these changes in peripheral CD4^+^ T cells could be employed as estimates of thymic output. CD31^+^ naive CD4^+^ T cells in the periphery are enriched in TCR excision circle (TREC^hi^) T cells (29) and therefore have been widely employed as a surrogate marker of recent thymic emigrants (RTEs) (30, 31). However, we found that quantification of CD31^+^CD4^+^ naive T cells as a proportion of total CD4^+^ T cells (referred as RTEs) provided better separation between thymic^+^ or thymic^–^ patients, showed stronger association with TREC values, and correlated well with DP cells in the thymus (Supplemental Figure 2, A–H).

With the validation of RTE percentage (RTE%) as a blood-based marker for thymic output, we expanded our study size (*n* = 110) with previously processed PBMCs from aged patients. Clinical characteristics are shown in Supplemental Table 3. Through linear regression analysis (Figure 2C), thymic output was shown to correlate positively with naive CD4^+^ T cells and negatively with central memory T (TCM) cells, consistent with our previous findings (Figure 2A). Similar correlations were observed with CD8^+^ T cells as well as effector memory T (TEM) cells that were previously unnoticed (Figure 2, A–C). However, these correlations were weaker, requiring a larger sample size (Figure 2C). Interestingly, no significant correlations were observed between RTE% in blood and effector memory RA^+^ T cells (TEMRA), which are reported to have a senescence-associated phenotype (Figure 2C) (32).

The regression of thymus size is not linear but phasic, with decreased decline later in life (33, 34). We next evaluated whether our cohort followed a similar pattern. Linear regression analysis produced no association between age and RTE% in patients aged over 50, while showing a high degree of heterogeneity in thymic output (Figure 2D). Under the age of 50 (clinical characteristics in Supplemental Table 4), when thymic activity declined at a faster rate, there was a significant negative correlation between RTE% in blood and chronological age (Figure 2C), supporting previous observations (8, 9). Interestingly, while the percentages of naive CD4^+^ and CD8^+^ T cells decline with age in younger individuals, this association was absent for CD4^+^ T cells in our older cohort, and only showed a weak association with CD8^+^ T cells (Supplemental Figure 2I).

The high heterogeneity observed among older individuals prompted us to investigate the factors influencing thymic output within this population with shared clinical confounders. Patient characteristics were compared via multiple regression analysis to RTE% and naive T cell percentage. Variables such as BMI, metabolic status, systemic inflammation, and cardiovascular diseases, including ischemia, had no association with RTE% (Supplemental Table 5). Interestingly, sex and smoking history were significantly associated with RTE% in contrast with naive T cell percentage, which fell above the cutoff threshold (Figure 2E). Levels of RTEs were significantly increased in females and for nonsmokers (Figure 2F). No statistical significance was observed between sex and smoking status in individuals under 50 years of age (Supplemental Figure 2J). These data indicate that sex and smoking can influence the rate of thymic atrophy in aged individuals.

### AT inflammation is independent of thymic output in aging.

**Figure 3:**
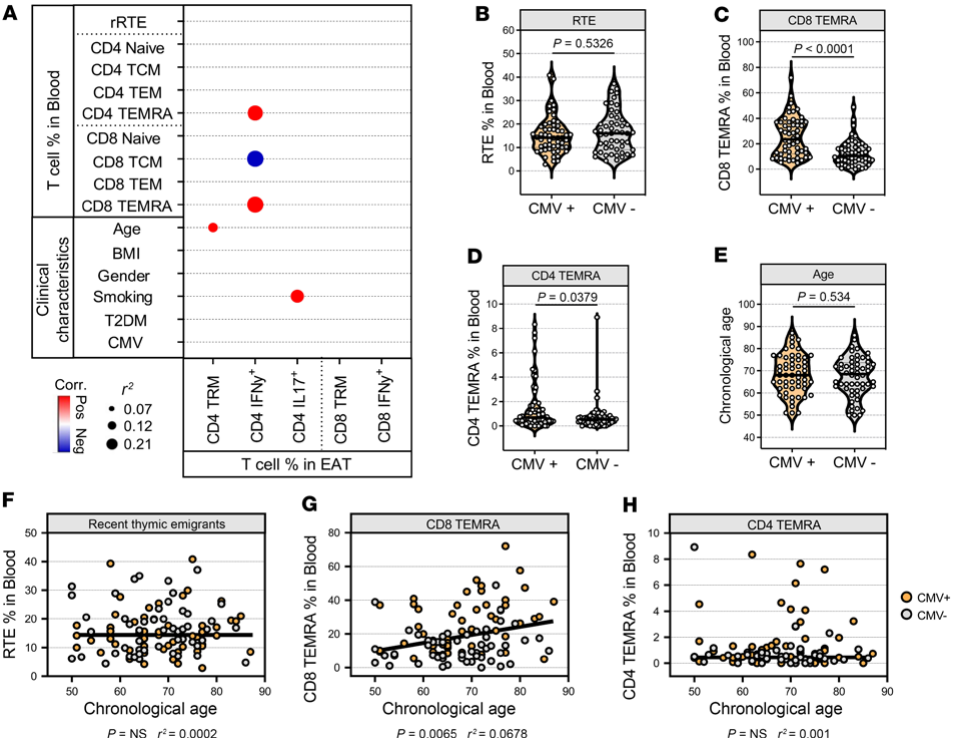
Adipose tissue inflammation in old patients is independent of thymic activity. (**A**) Bubble plot showing linear regression analysis comparing T cell percentage in EAT to blood T cell populations and clinical characteristics. Significant correlations (*P* < 0.05) are shown as red (positive correlation) or blue (negative correlation) dots. (**B**–**D**) Proportion of RTE, CD8^+^ TEMRA, and CD4^+^ TEMRA in the blood of CMV^+^ (*n* = 55) and CMV^–^ (*n* = 52) patients. (**E**) Comparison of chronological age between CMV^+^ and CMV^–^ patients. Violin plots show medians and interquartile ranges, and significance was evaluated by 2-sided Mann-Whitney *U* test. (**F**) Linear regression analysis between chronological age and proportion of RTE, (**G**) CD8^+^ TEMRA, and (**H**) CD4^+^ TEMRA in patients ≥50 years. The *r*^2^ values in **A** and **F**–**H** were calculated using Pearson’s correlation coefficients and significance by 2-sided *P* value analysis.

Early thymectomy has been associated with marked immunological changes reminiscent of an aged immune phenotype characterized by immunosenescence and an inflammatory state (17, 35–39). To explore the relationship between thymus output and tissue inflammation in our aged cohort, we assessed T cell subsets and T cell–mediated inflammatory cytokines in epicardial AT (EAT) and subcutaneous AT (SAT) by flow cytometry (Figure 3A and Supplemental Figure 3, A and B). EAT represents the fat situated between the visceral layer of the pericardium and myocardium and is commonly associated with a variety of cardiovascular/metabolic complications (40), while SAT has been associated with age-induced inflammation and senescence (41, 42). Through linear regression analysis, increasing age was found to associate with the percentage of CD4^+^ tissue-resident memory T (TRM) cells in EAT (*P* = 0.0224) (Figure 3A). Moreover, proportions of CD4^+^IFN-γ^+^ T cells were linked to increased blood CD4^+^ TEMRA (*P* = 0.0154, *r*^2^ = 0.1772) and CD8^+^ TEMRA cells (*P* = 0.0092, *r*^2^ = 0.2054) (Figure 3A), which are often utilized as a blood-based marker for immunosenescence (12, 32, 43). No significant correlations were found between inflammatory T cells in EAT and thymic output in our patients. Similar findings were observed in the SAT where CD4^+^IFN-γ^+^ T cells correlated positively with CD4^+^ TEMRA and TEM cells in blood, with no association being observed between RTE% and T cell inflammation (Supplemental Figure 3B–H). This suggests that peripheral factors, such as environmental and pathogen exposure, are associated with tissue inflammation. Indeed, we have previously found that EAT inflammation correlated with obesity and diabetes (40).

Altered CMV-related T cell profiles have been reported in a proportion of patients after thymectomy (17). As CMV has been shown to promote TEMRA differentiation, we next evaluated the impact of CMV infection in our aged cohort. Consistent with previous reports (39, 44, 45), our analysis revealed that CMV-positive patients exhibited elevated circulatory levels of both CD4^+^ and CD8^+^ TEMRA cells (Figure 3, C and D), with CD8^+^ fractions accumulating at a greater rate with increasing age (Figure 3, E, G, and H). However, we failed to detect any association with RTEs (Figure 3, B and F). Collectively, these findings indicate that tissue inflammation and T cell senescence are not necessarily associated with thymic function in old individuals.

### Thymic function is associated with a naive T cell signature.

**Figure 4:**
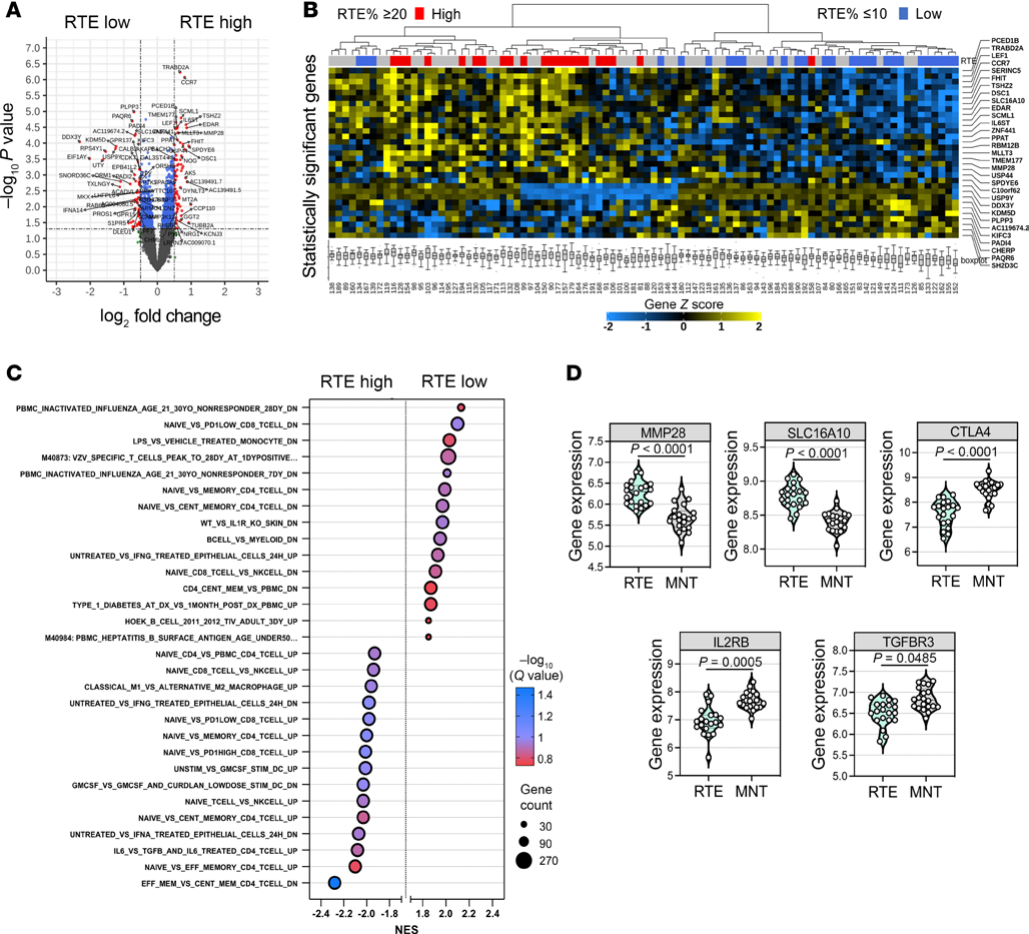
Evaluating transcriptional changes associated with thymic output in old age. (**A**) Volcano plot depicting differentially expressed genes between RTE-high and RTE-low patients. (**B**) Dendrogram with heatmap showing expression (*z* score) of the 30 statistically significant genes identified from RTE-high versus -low analysis applied to all patients (*n* = 94). Red and blue markers represent samples initially classified as RTE-high and RTE-low, respectively. (**C**) GSEA between patients grouped as RTE-low (RTE% ≤ 10) (*n* = 28) and RTE-high (RTE% ≥ 20) (*n* = 22). (**D**) Gene expression of *MMP28*, *SLC16A10*, *CTLA4*, *IL2RB*, and *TGFBR3* in human CD31^+^ (RTE) and CD31^–^ (MN) naive CD4^+^ T cells (*n* = 20). FDR < 0.05. Data presented as mean ± SD. Statistical significance evaluated by unpaired, 2-tailed Student’s *t* test.

To investigate whether increased thymic output could translate into changes in peripheral blood, the transcriptomic profile of whole blood samples from patients (*n* = 94) was assessed by microarray analysis. Principal component analysis did not reveal any major factor driving the variation in gene expression, with PC1 and PC2 only accounting for 6.63% and 4.65% of the variation, respectively (Supplemental Figure 4, A and B). To investigate genes that could associate with RTE%, we grouped patients into RTE-high (RTE% ≥ 20; *n* = 22) and RTE-low (RTE% ≤ 10; *n* = 28) based on mean RTE% between thymic^+^ and thymic^–^ patients (Supplemental Figure 2A). Differential gene expression analysis identified genes such as *CCR7*, *PCED1B*, *TRABD2A*, and *MMP28* among those upregulated in RTE-high versus -low samples (Figure 4A and Supplemental Table 6). With an FDR-adjusted *P* value of less than 0.1, statistically significant genes, when clustered, revealed good segregation between RTE-high and -low patients for the entire dataset (Figure 4B). Interestingly, most of these genes have been found to negatively correlate with age (46). Pathway analysis of gene sets associated with a naive T cell phenotype were enriched in RTE-high patients, whereas those related to exhaustion and TEM cells were enriched in RTE-low (significant enrichment at FDR < 25%) (Figure 4C, Supplemental Figure 4C, and Supplemental Tables 7 and 8).

To further explore the association of identified genes with naive T cells, we evaluated transcriptomic differences between CD31^–^ naive CD4^+^ T cells (MN T cells) and RTEs. Transcriptional analysis of publicly available microarray data from sorted human naive CD4^+^ T cells (47) showed an upregulation of genes linked to T cell activation (e.g., *IL2RB*, *CTLA4*) in MN T cells (Figure 4D). Genes linked with RTE phenotype identified from whole blood transcriptomic analysis (*MMP28* and *SLC16A10*) were differentially expressed between both cell groups. Notably, genes associated with naive CD4^+^ T cells in the context of aging, such as *TGFBR3* (48), were also identified among DEGs (FDR < 0.05). Gene set enrichment analysis (GSEA) revealed significant associations with T cell responses and T cell activation via IL-2/STAT5 signaling in MN T cells (Supplemental Figure 4D). With gene sets from GO biological processes, MN T cells had a noticeable enrichment for pathways linked to apoptosis and TGF-β production (Supplemental Figure 4E), which may be indicative of mechanisms associated with peripheral T cell homeostasis (49). Interestingly, RTEs were significantly enriched for oxidative phosphorylation, which could be an indication that these subsets retain a more quiescent T cell state in circulation (50, 51).

### Thymic function is important for a broad TCR repertoire diversity.

**Figure 5:**
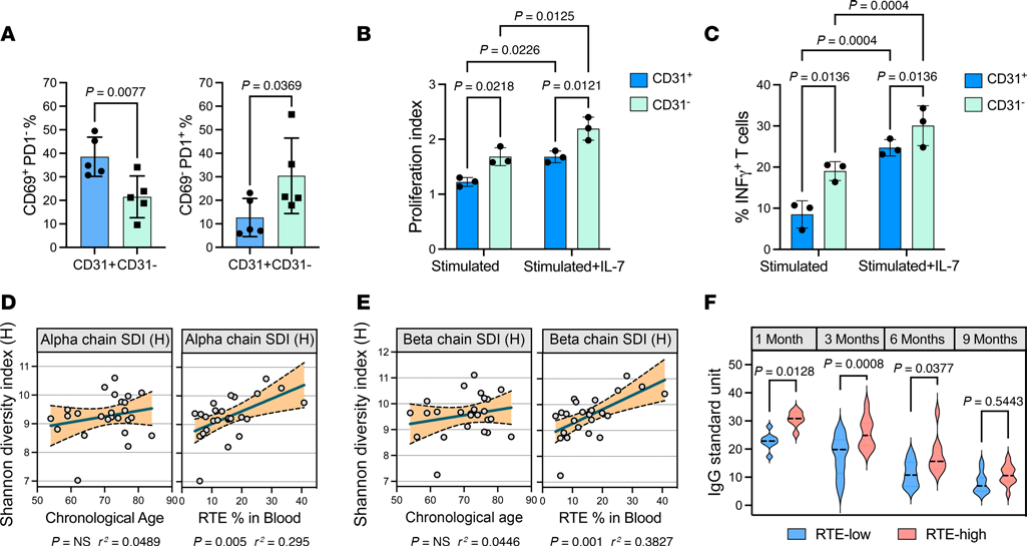
Preserved thymic activity in old patients promotes TCR repertoire diversity. (**A**) Graphs showing CD69^+^PD-1^–^ and CD69^–^PD-1^+^ T cells 5 days after stimulation. Statistical significance evaluated by paired, 2-tailed Student’s *t* test (*n* = 5). (**B**) Proliferation index calculated via CFSE fluorescence cocultures with or without IL-7. (**C**) Graph showing the proportion of IFN-γ^+^ T cells cocultured with or without IL-7. Data in **A**–**C** presented as mean ± SD; significance between groups evaluated by 2-way ANOVA with Tukey’s test for multiple comparisons (*n* = 3). (**D**) Shannon diversity index (SDI) values for α and (**E**) β TCR chains correlated to chronological age and RTE% (*n* = 25). The *r*^2^ values in **D** and **E** were calculated using univariate Pearson’s correlation coefficients and significance by 2-sided *P* value analysis. (**F**) Graph showing influenza A–specific IgG antibody levels in patients 1 (*n* = 7–8), 3 (*n* = 12–11), 6 (*n* = 8–9), and 9 months (*n* = 11–12) after immunization. Data presented as mean ± SD. Statistical significance evaluated by 2-way ANOVA with Šídák’s multiple-comparison test.

The thymus is critical for the normal development of the immune system in early life. Thymectomy during childhood results in reduced T cell counts, increased oligoclonal memory, and decreased cellular immunity (17, 36). The health benefits to adults are less clear, considering the thymus naturally regresses with age (8). To explore the immunological impact of thymus function and changes in naive T cell populations in older age, we performed functional activation assays using sorted RTEs and MN T cells from humans. Naive T cells were cocultured with antigen-presenting cells (APCs) in the presence of a synthetic superantigen (CytoStim). After 5 days of culture in vitro, MN T cells had increased cell surface programmed cell death protein 1 (PD-1) expression, which was accompanied by greater proliferation and production of IFN-γ compared with RTEs (Figure 5, A–C, and Supplemental Figure 5, A–E). Notably, when cultured with IL-7, both subsets demonstrated increased proliferation and cytokine production (Figure 5, B and C). Collectively, these findings suggest that MN T cells, despite sharing similar naive surface markers with RTEs, exhibit distinct functional characteristics, including heightened responsiveness to stimulation and enhanced cytokine production. Indeed, RTEs are phenotypically and functionally immature and therefore are believed to undergo postthymic maturation in the periphery, transitioning to a more functional MN T cell state (52, 53).

Although CD31^+^ naive T cells undergo turnover to a certain degree, they are enriched in RTEs with a high degree of TCR diversity (29). Thus, to expand our understanding into the immunological impact of thymic activity in aged individuals, we conducted TCR α and β chain sequencing to evaluate TCR repertoire diversity. Shannon diversity index (H) calculations revealed significant correlations between RTE%, but not age, in blood for both α and β chains (Figure 5, D and E). Further analysis showed significant positive correlations with RTE% and TCR α chain CDR3s matched to known antigens (McPAS-TCR database) associated with pathogens (*P* = 0.0483, *r*^2^ = 0.159), cancer (*P* = 0.0175, *r*^2^ = 0.222), and allergy (*P* = 0.0316, *r*^2^ = 0.186), which is indicative of higher de novo diversity within CDR3 (54, 55) (Supplemental Figure 5F).

To test the relevance of a diverse TCR repertoire in ensuring effective immune responses, we evaluated the antibody response to the annual influenza vaccine in our patient cohort. Patients were grouped based on time after vaccination and RTE-low or -high. Both groups showed decreased IgG antibody titers over time, with RTE-high patients showing increased antibody response to vaccination (Figure 5F). Similarly, we found that patients with maintained thymic function were less likely to develop a lower respiratory tract infection during intensive care unit stay (Supplemental Table 9). Collectively, these data show the relevance of thymic output for efficient protection against pathogen infections.

### Low thymic output is associated with accelerated epigenetic aging.

**Figure 6:**
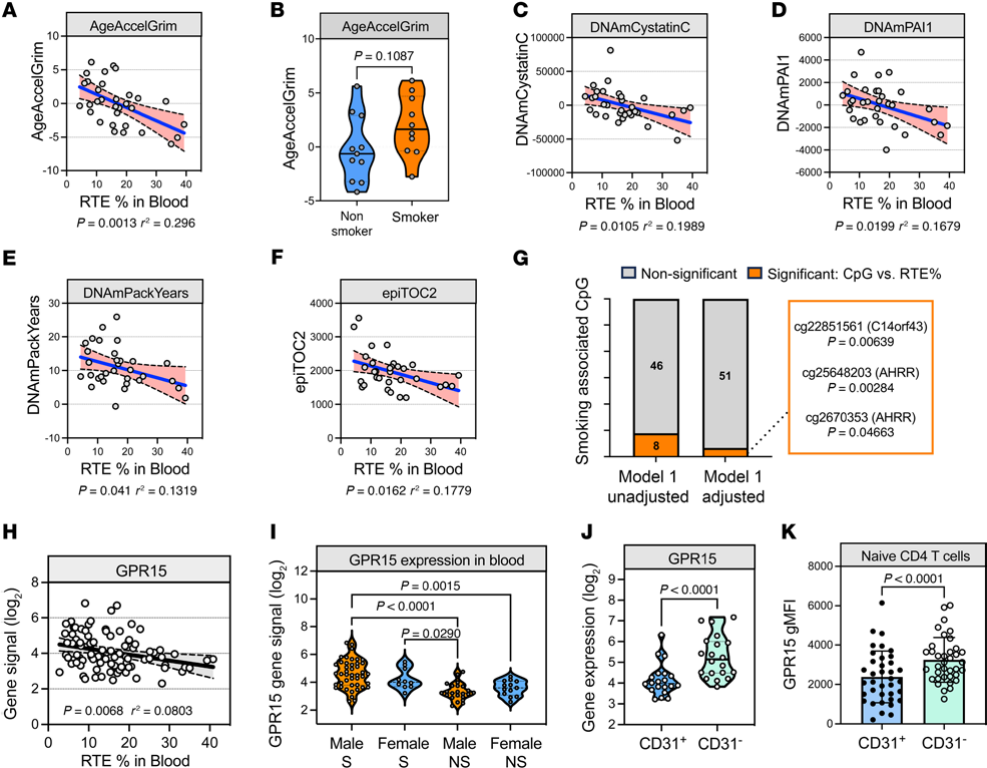
Reduced thymic activity associates with epigenetic age acceleration in old patients. (**A**) GrimAge age acceleration (AgeAccelGrim) against RTE% in blood (*n* = 32). (**B**) AgeAccelGrim values compared between male nonsmokers (*n* = 11) and smokers (*n* = 10). Truncated violin plots show medians and interquartile ranges, and significance was evaluated by unpaired, 2-tailed Student’s *t* test. (**C**) Correlation between RTE% in blood and DNAm surrogates for cystatin C, (**D**) plasminogen activator inhibitor 1 (PAI-1), (**E**) smoking (PackYears), and (**F**) mitotic age (epiTOC2). (**G**) Significant (orange) and nonsignificant (gray) smoking-associated CpGs correlating with RTE%. Model 1: No covariates; Model 2: age, sex, and major blood cell type DNAm estimates (monocytes, B cells, neutrophils, CD4^+^ T cells, NK cells). (**H**) Linear regression between RTE% and *GPR15* gene expression from whole blood microarray data. (**I**) *GRP15* gene expression from whole blood between patient subgroups defined by sex and smoking status. Violin plots show medians and interquartile ranges, and significance was evaluated by 1-way ANOVA with Tukey’s test for multiple comparisons. Only significant values are shown. S, smoker; NS, nonsmoker. (**J**) *GPR15* gene expression (*n* = 20) and (**K**) geometric mean fluorescence intensity (gMFI) of GPR15 protein expression from CD31^+^ and CD31^–^ naive CD4^+^ T cells (*n* = 37). Data presented as mean ± SD; significance by paired, 2-tailed Student’s *t* test. The *r*^2^ values in **A**, **C**–**F**, and **H** were calculated using Pearson’s correlation coefficients and significance by 2-sided *P* value analysis.

Our previous data suggest that contraction of the TCR repertoire may hinder immune protection against infections. To investigate whether natural involution of the thymus in old age associates with increased risk of mortality, we investigated the correlation between RTEs and epigenetic DNA methylation (DNAm) clocks, which are widely used to quantify and predict “biological” age and mortality risk. The DNAm GrimAge (AgeAccelGrim) represents a recent and refined second generation “phenotypic” clock that, in addition to DNAm estimates of age, incorporates estimators of 7 aging-related plasma proteins and pack-years smoking exposure (DNAmPack) (56), thereby improving the prediction of morbidity and mortality to outperform first-generation Hannum and Horvath’s DNAm clocks (57). We found a significant negative correlation between RTE% and age acceleration values from GrimAge (Figure 6A and Supplemental Figure 6). No significant differences were observed between smoking status and AgeAccelGrim values (Figure 6B). The association of AgeAccelGrimAge with RTE% was attributed to a combined effect of DNAm surrogates for cystatin C (DNAmCystatinC) (Figure 6C), plasminogen activator inhibitor 1 (PAI-1) (DNAmPAI1) (Figure 6D), and smoking (DNAmPackYears) (Figure 6E), which are all components of GrimAge. The DNAm estimate of mitotic age (epiTOC2) (58), indicative of cumulative cell division and cancer risk, was also shown to correlate with thymic activity in our cohort (Figure 6F). Together, these data support the notion that continued thymic function is associated with reduced mortality.

Transcriptomic and epigenomic studies have shown shifts from naive to differentiated effector/memory T cell states in smokers (59, 60). Prior studies have identified thousands of DNAm changes associated with smoking in peripheral blood, of which 62 have been widely reported across multiple studies (61). To investigate whether certain changes could be associated with thymus function in elderly individuals, we evaluated DNAm array–derived β values for known smoking-associated CpGs against RTE% in patient blood through linear regression analysis. Of the 62 CpGs, 54 are present on the Illumina EPIC (850k) array, from which 8 were significantly associated with thymic output: cg21121843 (*HTT*); cg26703534, cg25648203 (*AHRR*); cg01731783, cg22851561 (*C14orf43*); cg12803068 (*MYO1G*); cg06060868 (*SDHA*); and cg20295214 (*AVPR1B*) (Figure 6G). While smoking-related changes in cell proportions contribute to the DNAm signature observed (61), 3 of these results, cg25648203 (*AHRR*), cg26703534 (*AHRR*), and cg22851561 (*C14orf43*), were still significant after adjusting for major blood cell type proportions and additional covariates (Figure 6G). Changes in DNA methylation and gene expression of *GPR15* have been strongly associated with smoking status (59, 62). Although methylation at CpG site cg19859270 of *GPR15* failed to reach significance, the gene expression signal for *GPR15* correlated negatively with RTE% (*P* = 0.0068, *r*^2^ = 0.0803) (Figure 6H) and was elevated in smokers (Figure 6I). Furthermore, GPR15 expression was significantly higher in naive CD31^–^CD4^+^ T cells compared with naive CD31^+^CD4^+^ T cells at the protein and RNA level (Figure 6, J and K). Indeed, hypomethylation at the cg19859270 site in smokers is believed to be the result of changes in the proportion of T cell populations in the peripheral blood (63). Therefore, our data on GPR15 expression support a link between smoking and thymic output.

## Discussion

With global populations aging at greater rates and declining immunological proficiency being the subject of numerous clinical studies, substantial efforts have been made toward understanding the factors influencing the immune system with increasing age (64). Among these factors, thymic involution, a process that impedes the production of new T cells, is thought to contribute to the process of immune aging. The immune system undergoes a myriad of changes as age advances, but less is known about how thymus involution contributes to the dysregulated immunity observed in older age. In this study, we aimed to investigate factors influencing thymic output and thymus-associated health outcomes in individuals 50 years of age or older. Our findings reveal the persistence of functional thymic structures in ATs from within the mediastinal cavity, that were influenced by factors such as sex and smoking status. These structures possess the capacity to facilitate T cell development in old age contributing to improved immune surveillance.

In support of previous studies, we show that greater thymic output correlates with increased naive T cells and decreased memory T cells for both CD4^+^ and CD8^+^ subsets in the periphery. Chronological age, as opposed to sex differences and smoking status, had no influence on thymopoiesis in our cohort of patients, which aligns with previous reports that have shown a deceleration in thymic involution after adulthood (31, 65). Differences in sex were readily evident at the tissue level, with 89% of females being thymic^+^, which is consistent with previous observations (66). Recent work by Marquez et al. has revealed that sexual differences in human immune responses are more pronounced among older individuals, with males displaying a greater decline in naive T cell activity compared with age-matched females (67). Sex-associated hormones such as androgens are known to negatively regulate thymic stromal populations through pathways that impair the survival and differentiation of thymocytes (68–70). Furthermore, the increased male bias in diseases associated with impaired immune responses (71) may be partially influenced by thymus functionality.

The damaging effects of smoking and its association with various pathophysiological complications have been well characterized over the years. Through increased proinflammatory responses, oxidative stress, and DNA damage, smoking can severely restrict both innate and adaptive immune responses (72–74). The noticeable decrease in RTEs observed in smokers, regardless of sex, shows a potential association between thymus proficiency and smoking status. Of note, reporting smoking status in a 12-month period may weaken association studies, as it does not consider packs per year and duration. Whether smoking leads to diminished thymus function directly or indirectly through immunological changes is unclear. Recent studies have found that smoking influences the expression and methylation of GPR15, with increased expression of GPR15 on T cells being linked to smoking-induced systemic inflammation (63, 75). We found that *GPR15* gene expression correlated significantly with the proportions of RTEs in blood. This could be partially explained by high expression of GPR15 on CD31^–^ compared with CD31^+^ naive T cells. The functional role of GPR15 remains elusive, but it has been implicated in the migration of lymphocytes to large intestinal mucosa to maintain homeostatic conditions during inflammation in mice (76, 77). Additional research is required to further understand the role of GPR15 in T cell–mediated responses beyond its role as a valuable blood-based marker for smoking-induced immune changes.

The evolutionary significance of thymus involution has been widely stipulated, as a loss of thymic activity can lead to the development and progression of various disease states. However, it is only recently that the importance of the thymus in adult life has been reported. In a similar cohort of patients, Kooshesh et al. found that removal of the thymus increases all-cause mortality while propagating risk for cancer and autoimmune diseases (10). However, it is unclear whether these observations hold true during natural thymic involution. We found that RTE% negatively associated with DNAm GrimAge, a mortality risk estimator. The GrimAge clock was constructed with the addition of 7 DNA methylation–based markers for plasma proteins and smoking packyears (66), which are related to many age-related conditions. In addition to smoking, we found that RTE% associated with the 2 plasma proteins, PAI-1 and cystatin C. PAI-1 plays a central role in several age-related subclinical and clinical conditions and has been linked to lifespan in genetic studies (78). Plasma cystatin C is related to many age-related traits and has been associated with unsuccessful aging (79, 80). Cystatin C is suggested to regulate DC function and cathepsin S activity, while PAI-1 was shown to promote PD-L1 expression, which could have an impact on immunity (81–83). In addition, individuals with greater thymic output have lower mitotic count (epiTOC2) (58), which is associated with increased cancer risk, independent of chronological age. The less diverse TCR repertoire in individuals with low RTE% could conceivably contribute to the immune escape and development of cancer, while cystatin C and PAI-1 could have an unforeseen role in promoting cancer risk in these patients (84, 85).

Despite showing that thymic activity in old age influences naive/memory peripheral T cell pools and TCR repertoire diversity, we failed to observe differences in inflammaging and immunosenescence in our patient cohort. Thymic output associates with a higher pool of naive T cells with an enriched fraction of CD31^+^ T cells, with CD31^–^ naive T cells having greater proliferative capacity, activation-induced PD-1 expression, and cytokine production (IFN-γ) when compared with RTEs. It is worth noting that PD-1 has been shown to regulate memory T cell differentiation and to limit the generation of TCM cells, which will have a negative impact on long-term adaptive memory (86). In addition, greater α/β chain diversity as a result of preserved thymic output would offer improved pathogen recognition and response to a greater variety of novel antigens outside of the preexisting memory T cell population, as has been shown for influenza vaccine efficacy (87, 88). In line with this, we found that patients with reduced thymic output had lower antibody responses to seasonal influenza vaccine and were more likely to develop hospital-acquired respiratory infections after surgery.

As this study includes patients undergoing cardiac surgery, which allowed the isolation of tissue samples from within the chest cavity, extrapolation of our finding to healthy aged individuals should be done with caution. However, similar sex differences in thymic output have been observed (66), while smoking has been associated with lower thymic CT score in the general population (89). Furthermore, our study found no association with systemic inflammation (e.g., C-reactive protein) or type of surgery. No differences were observed between ischemic (coronary artery bypass grafting, CABG) or valve replacement patients with no heart failure, suggesting that the observed thymic output heterogeneity is not restricted to our cohort of patients. In addition, a decrease in the frequency of naive T cells and TCR repertoire diversity, which are hallmarks of thymic function, have been associated with frailty in advanced age (90, 91). This suggests a degree of heterogeneity in thymus function during aging across the general population that will have immunological consequences beyond the established aspects of chronological age.

## Methods

### Sex as a biological variable.

This study examined both females and male patients and sex-dimorphic effects are reported.

### Study population and sample collection.

Adult patients, undergoing on-pump open-chest CABG surgery, valve surgery (CSS angina class 0 to 1 and a NYHA class 1 to 2), or combined CABG/valve surgery were recruited from Barts Heart Centre, St. Bartholomew’s Hospital via the Barts BioResource. An additional cohort under 50 years of age was recruited through the clinic at Barts Heart Centre. Patients were screened and then informed written consent was taken for study participation as per local research procedures and Good Clinical Practice guidance. Exclusion criteria included patients with congenital heart disease, underlying cardiomyopathies or ion channelopathies, primarily undergoing other cardiac surgical procedures (e.g., aortic surgery), off-pump CABG surgery, patients with active endocarditis, myocarditis, or pericarditis, those with preexisting inflammatory diseases such as rheumatoid arthritis, active malignancy, patients on immunomodulatory or biologic drugs (such as tacrolimus or anti–TNF-α agents), perioperative rhythm control therapies (e.g., use of amiodarone), postoperative hemodynamic shock, uncorrected potassium derangement (K < 3.3 or K > 5.8), or uncorrected magnesium derangement (Mg < 0.5 or Mg > 1.5) detected on laboratory blood sample analysis. Fasting venous blood samples were collected preoperatively in the anesthetic room. A current smoker was defined as an individual with a recent smoking status in the 12 months preceding surgery as per NHS guidelines. Patients were monitored after surgery during their stay in the intensive care unit. Blood test results, cardiac monitoring, and complications were recorded on a daily basis. Approximately 0.8–1 g of AT samples were collected in ice-cold phosphate-buffered saline with 2% fetal bovine serum. SAT was collected immediately following the median sternotomy incision and EAT was obtained following opening up of the pericardial sac.

### Blood and AT sample processing.

Fasting blood samples were collected preoperatively to include 6.5 mL of peripheral blood divided into 2.5 mL collected in a PAXgene (PreAnalytiX) tube for RNA isolation and the remaining 4 mL in an EDTA tube (BD). PBMCs were isolated using Ficoll-Paque PLUS (GE Healthcare, now Cytiva) as per the manufacturer’s instructions. PBMCs were then stained using antibodies for flow cytometry analysis. The gating strategy is depicted in Supplemental Figure 1. Following AT sample collection, samples were divided into a portion for flow cytometry (~0.1–0.4 g) analysis; a portion for subsequent RNA extraction (~0.1–0.2 g), which was snap-frozen; and a sample fixed in 4% formaldehyde solution (MilliporeSigma) for future immunohistochemical analysis (~0.05–0.2 g). The sample of AT aliquoted for flow cytometry analysis was first mechanically minced using microscopy scissors and then digested enzymatically using 5668 IU collagenase II (MilliporeSigma) and 55.5 IU DNase (MilliporeSigma) per gram of AT for 30 minutes. Immune cells present in the stromal vascular fraction were obtained following centrifugation and lysed for red blood cells prior to antibody staining.

### Flow cytometry staining and analysis.

Immune cells were stained with fixable Aqua LIVE/DEAD cell stain (Invitrogen) diluted 1:1000 and fluorochrome-conjugated antibodies specific for CD197-FITC (BioLegend, 353216; 1:200 dilution), CD19-PerCP-Cy5.5 (BioLegend, 302228; 1:200 dilution), CD45RO-BV421 (BioLegend, 304224; 1:200 dilution), CD335-BV605 (BioLegend, 331926; 1:200 dilution), CD45-BV785 (BioLegend, 304048; 1:200 dilution), CD127-APC (BioLegend, 351342; 1:200 dilution), CD8-AF700 (BioLegend, 300920; 1:500 dilution), CD3-APC/Cy7 (BioLegend, 300318; 1:200 dilution), CD69-PE (BioLegend, 310906; 1:200 dilution), CD4-PE/Cy7 (BioLegend, 357410; 1:200 dilution), PD-1-PE-CF594 (BioLegend, 329940; 1:200 dilution), KLRG1-SB702 (eBioscience, 15824202; 1:200 dilution), CD303-FITC (BioLegend, 354208; 1:200 dilution), CD123-PerCP/Cy5.5 (BioLegend, 306016; 1:200 dilution), CD206-BV421 (BioLegend, 321126; 1:200 dilution), CD3-BV605 (BioLegend, 317322; 1:200 dilution), CD19-BV605 (BioLegend, 302244; 1:200 dilution), CD14-APC (BioLegend, 325608; 1:200 dilution), CD16-AF700 (BioLegend, 302026; 1:500 dilution), CD1c-APC/Cy7 (BioLegend, 331520; 1:200 dilution), Clec9A-PE (BioLegend, 353804; 1:200 dilution), CD1a-PE-CF594 (BioLegend, 300132; 1:200 dilution), and CD141-PE/Cy7 (BioLegend, 344110; 1:200 dilution). The samples were stained at 4°C for 18 minutes, washed twice with FACS buffer, and fixed in stabilizing fixative buffer (BD) containing 3% paraformaldehyde at 4°C for 30 minutes. For intracellular cytokine staining, samples were resuspended in 500 μL AIM V medium Thermo Fisher Scientific with the addition of 1 uL of Cell Activation Cocktail (BioLegend). After 4 hours of incubation, samples were washed and stained for surface markers as detailed above followed by permeabilization and fixation in the permeabilization/fixation buffer (BD) at 4°C for 12 minutes. Intracellular cytokine production was evaluated by incubation 15 minutes at 4°C with fluorochrome-conjugated antibodies specific for IFN-γ-APC (BioLegend, 502511; 1:200 dilution), IL-17-APC/Cy7 (BioLegend, 512319; 1:200 dilution), and IL-22-PE (BioLegend, 366703, 1:200 dilution). Data were acquired on a Cytoflex (Beckman Coulter) and analyzed using FlowJo version 10 software. For quantitative analysis of thymic structures, number of cells were normalized to gram of tissue. This is, however, an estimate influenced by experimental factors such as cell recovery and tissue digestion efficacy.

### RNA extraction.

Total RNA from patient blood samples was extracted using a PAXgene blood RNA kit (PreAnalytiX). In brief, 2.5 mL of blood was collected into PAXgene RNA tubes and incubated at room temperature for 2 hours. Blood samples were then centrifuged for 10 minutes at 3000*g*, and supernatants were discarded by decanting. Residual pellets were stored at –80°C for later processing or total RNA was extracted following the manufacturer’s instructions immediately. To extract RNA from ATs, samples were finely minced using sterile scissors treated with RNAlater (Invitrogen). To the homogenized tissues, 1 mL QIAzol lysis reagent (Qiagen) was added and thoroughly mixed through inversion. Samples were left at room temperature for 5 minutes and then 200 μL chloroform was added. Total RNA was extracted using RNeasy Lipid Tissue Mini kits (Qiagen) following the manufacturer’s instructions. Relative yields for blood and tissue RNA were quantified using a NanoDrop spectrophotometer.

### RT-PCR and bulk RNA-seq.

Reverse transcription to cDNA was performed using High-Capacity RNA-to-cDNA kits (Applied Biosystems, Thermo Fisher Scientific) and stored at –80°C. The relevant primer sequences were purchased from Invitrogen, Thermo Fisher Scientific. Gene expression was performed using SYBR Green Supermix (Bio-Rad), as per the manufacturer’s instructions, and analyzed using the Light Cycler System (Roche). Relative gene expression values were determined using the ΔΔCt method and normalized to a stable reference housekeeping gene control (*GAPDH*). Illumina sequencing for bulk RNA-seq was carried out at Novogene Bioinformatics Technology Ltd.

### TREC quantification.

Thymic function was evaluated indirectly by measuring single joint TREC values in whole blood as previously described (92), with some modifications. Briefly, sjTREC and TRAC fragments were amplified and purified TREC and TRAC PCR products were cloned into the pCR 2.1 vector using a TA Cloning kit (Thermo Fisher Scientific). Cloned fragments were used to create a standard curve. gDNA was extracted from 200 μL of whole blood and resuspended in 200 mL for qRT-PCR analysis. Two milliliters of sample was initially amplified using sjTREC and TRAC primers for 20 cycles to guarantee an accurate amplification via RT-PCR (93). A further 2 mL of a 1:10 dilution from the first round of PCR amplification was then amplified via qRT-PCR reaction consisting of 2 μL gDNA, 500 μM primers, and 1× iTaq Universal SYBR Green, before being analyzed using a CFX Connect light cycler. The copies of sjTRC and TRAC were calculated using a standard curve. The numbers of TRECs were calculated with the following formula: mean quantity TRECs/(mean quantity TRAC/2) × 500 = copies/mL.

### Gene microarray.

One hundred nanograms of total RNA extracted from whole blood was submitted to the University of Bristol Genomics Facility for whole transcriptome expression analysis with the Clariom S Array Plate platform using the GeneChip WT Plus Reagent Kit, following the GeneChip WT Plus user guide (catalog numbers 902280 and 902281). Transcriptome analysis Suite v4.0.2.15 was used to perform normalization and summarization of the array pegs (100). The raw data have been deposited in the NCBI Gene Expression Omnibus (GEO GSE261587).

### Methylation microarray and epigenetic clock models.

In brief, genomic DNA was extracted from whole blood using the QIAamp DNA Blood Mini kit (Qiagen). DNA quality was assessed with an Agilent TapeStation. Thirty-two DNA samples were then submitted to University of Bristol Genomics Facility for bisulphite conversion (Zymo Kit) (94) and hybridization on Infinium MethylationEPIC (v1) BeadChip Arrays via the Illumina iScan System using the manufacturer’s standard protocol. Raw iDAT files were obtained and standard quality control (QC) and preprocessing was performed via the R package meffil (95). All samples passed QC metrics for correct sex prediction, median methylation intensity, control probe outliers (staining, extension, target removal, hybridization, bisulphite conversion), sample detection (probes with a detection *P* value between 0.01 and 0.1), and proportion of probes over bead threshold (0.1 < bead number < 3). The DNAm smoking association analysis with RTEs was performed via meffil (95) with 2 regression analysis models — (a) no covariates and (b) covariates, including age, sex, major blood cell types, and DNAm estimates (monocytes, B cells, neutrophils, CD4^+^ T cells, NK cells) (96), in addition to surrogate variables. The 54 available Illumina Epic array results from the Gao et al. strongly replicated smoking-associated CpGs (61) were extracted to identify those passing nominal significance (*P* ≤ 0.05). Then for DNAm clock analysis, the minfi R package (97) was used to implement the recommended probe-type normalization (preprocessNoob). A subset of the 30,084 CpGs was extracted from the total set using the datMiniAnnotation3.csv file for advanced analysis using the online Horvath lab DNAm age calculator (https://dnamage.genetics.ucla.edu/) (98). Due to differences in the 850k array from earlier 450k/27k arrays, 2,552 CpGs are not included in this list. The methylCIPHER package was also used to calculate additional DNAm clocks plus DNAm trait estimators (99). These included the blood-derived GrimAge (56) and mitotic EpiTOC2 (58) DNAm clocks.

### TCR sequencing.

TCR α and β chain sequencing was performed by using RNA extracted from whole blood and employing a quantitative experimental and computational TCR sequencing pipeline, as previously described (100). The pipeline introduces unique molecular identifiers (UMIs) attached to individual cDNA TCR molecules, allowing correction for PCR and sequencing errors. TCR identification, error correction, and CDR3 extraction were performed following a suite of tools available at https://github.com/innate2adaptive/Decombinator, as detailed previously (101). Bulk TCR-seq data are available at Zenodo (https://doi.org/10.5281/zenodo.15097049).

### T cell stimulation assays and antibody concentration measurements.

In brief, naive CD4^+^ T cells were negatively isolated from LSR cones obtained from the NHS using the EasySep Human Naive CD4 T cell isolation kit (STEMCELL Technologies, 19555). Purified cells were stained with Aqua and CD31-APC (BioLegend, 303115; 1:200 dilution), CD4-PE/Cy7 (BioLegend, 357410; 1:200 dilution), and CD8-AF700 (BioLegend, 300920; 1:500 dilution) surface antibodies at 4°C for 18 minutes and then washed twice with FACS buffer. Live CD4^+^CD31^+^, CD4^+^CD31^–^, and CD8^+^ cells were separated by FACS with an LSRFortessa analyzer (BD). APCs were positively isolated from matched PBMC samples using Biotin anti-human HLA-DR antibody (BioLegend, 307614; 1:200 dilution) and MojoSort Streptavidin Nanobeads (BioLegend, 490016). Sorted naive T cells and APCs were cocultured at a ratio of 5:1 in AIM V media supplemented with 20 U/mL IL-2 with or without CytoStim (Miltenyi Biotec). For proliferation assays, naive T cells were labeled with 3 μM CFSE (Life Technology, C1157) prior to stimulation. After 5 days of culture at 37°C and 5% CO_2_, cells were stained for intracellular cytokines, as described above.

### Determination of antibody levels by ELISA.

Plasma samples from patients were collected on the day of surgery. To evaluate CMV serostatus in patients, CMV-specific IgG antibodies were detected via ELISA. Similarly, anti–influenza A IgG concentration was measured using a commercially available kit from Abcam according to the manufacturer’s instructions. Briefly, plasma samples were diluted 1:100 and incubated for 1 hour on 96-well microtiter plates coated with influenza nucleocapsid and envelope proteins. Plates were washed and incubated with HRP-conjugated anti-human antibodies and detected using TMB substrate. Optical density was analyzed using a spectrophotometric plate reader (BMG Labtech) at 450 nm wavelength absorbance. Samples were included if their absorbance values were greater than the cutoff threshold by more than 10%.

### Statistics.

Sample size calculations were based on the correlation of RTE% and naive CD4^+^ and CD8^+^ T cells, with naive CD8^+^ T given the higher sample size of 36 patients with 90% power (α = 0.05, effector size = 0.489). Statistical significance was determined for continuous variables using a 2-tailed Student’s *t* test when the data were parametric and the Mann-Whitney *U* test for nonparametric data. Spearman’s correlation was used to analyze univariate associations between quantitative variables. Logistic regression was utilized to calculate *r*^2^ values between continuous variables. The χ^2^ test or Fisher’s exact test was utilized for categorical data. Data were analyzed using Prism version 9 (GraphPad Software LLC). Normality was assessed using the Kolmogorov-Smirnov and Shapiro-Wilk tests. Where parametric data are represented, the mean and SD values are reported, and for nonparametric data, median and interquartile ranges are reported.

### Study approval.

All patients provided written informed consent for their participation in the study as per local research procedures and Good Clinical Practice guidance. Barts BioResource has been approved by the East of England-Cambridge Central Research Ethics Committee (reference: 14/EE/0007).

### Data availability.

RNA-seq raw and processed data are deposited in the NCBI GEO under accession number GSE261587. Bulk TCR-seq data are available at Zenodo (https://doi.org/10.5281/zenodo.15097049). The suite of tools for TCR-seq analysis can be accessed at https://github.com/innate2adaptive/Decombinator DNA methylation data have been deposited in the NCBI GEO under accession number GSE296328. Additional data generated in this study are provided in the Supporting Data Values file.

## Author contributions

The manuscript was initially drafted by BS and MPL reviewed and edited the manuscript. BS and MPL contributed to study design and development of the concept. BS made substantial contributions to data acquisition and analysis. VV and DH recruited patients and provided clinical data. FMMB, PB, and RR contributed with resources and interpretation of data. CGB and BMC analyzed DNAm and TCR-seq data, respectively. All authors contributed to critical review and revision of the manuscript.

## Supplementary Material

Supplemental data

ICMJE disclosure forms

Supplemental tables 2, 6, 7 and 8

Supporting data values

## Version 1 07/01/2025

In-Press Preview

## Version 2 08/08/2025

Electronic publication
