# Supplementary material for: Heterogeneity of thymic output in the elderly and its association with sex and smoking
Source: JCI Insight. 2025 Jul 1;10(15):e189008. doi: 10.1172/jci.insight.189008 (PMC12341542; doi:10.1172/jci.insight.189008)
Supplement: Supplemental data [file jciinsight-10-189008-s042.pdf]

Supplementary File

<u>Heterogeneity of thymic output in elderly and its association with sex and smoking.</u>

Balraj Sandhar[1], Vishal Vyas[1, 2], Daniel Harding[1, 2], Roberta Ragazzini [3, 4], Paola Bonfanti [3, 4], Federica M. Marelli-Berg[1], Christopher G. Bell[1], Benny Chain[5], M. Paula Longhi[1].

[1] William Harvey Research Institute, Barts and The London School of Medicine and Dentistry, Queen Mary University of London, London, United Kingdom.

[2] Department of Cardiology, Barts Heart Centre, St Bartholomew's Hospital, London, United Kingdom.

[3] Epithelial Stem Cell Biology & Regenerative Medicine laboratory, The Francis Crick Institute, 1 Midland Road, London NW1 1AT, UK

[4] Institute of Immunity & Transplantation, Division of Infection & Immunity, UCL, Pears Building, Rosslyn Hill, London NW3 2PP, UK

[5] Division of Infection and Immunity, University College London, London, United Kingdom.

Correspondence:
M. Paula Longhi
Address: Heart Centre, William Harvey Research Institute, Barts and The London School of Medicine and Dentistry, Queen Mary University of London, Charterhouse Square, London, EC1M 6BQ
Fax:
Tel: +44 (0) 207 882 6566
Email: m.longhi@qmul.ac.uk

Supplementary Figure 1. Profiling CD45$^+$ cell subsets and cortical/medullary structures in thymic tissue. A) Flow cytometry gating strategy for the identification of different lymphoid and B) myeloid subsets from mediastinal adipose tissue samples. C) Immunofluorescent images characterising thymic cortical and medullary epithelial compartments through the differential expression of cytokeratin 5 and 8 in DP T cell$^+$ mediastinal adipose tissue. D) Identification of AIRE$^+$ medullary thymic epithelial cells in DP T cell$^+$ mediastinal adipose tissue. E) Gene set enrichment analysis with KEGG, GOBP, and ImmuneSigDB gene sets between thymic$^+$ (n = 3) and thymic$^-$ (n = 4) samples. Plots depict either most enriched, significant or relevant gene sets.

Supplementary Figure 2. Assessing peripheral markers for thymic activity. A) Proportion of recent thymic emigrants in blood of thymic$^+$ and thymic$^-$ patients (thymic$^+$, n = 27; thymic$^-$, n = 19). B) Proportion of CD31$^+$ naïve CD4 T cells in the blood of thymic$^+$ (n=27) and thymic- (n=19) patients. C) TREC copies per ml of blood in thymic$^+$ (n=14) and thymic$^-$ (n=19) patients. Error bars on graph represent mean ± SD. Statistical significance evaluated by unpaired two-tailed t test. A-C) Error bars on graph represent mean ± SD; significance evaluated by unpaired two-tailed t test. D) Linear regression showing relationship between both metrics used for gauging peripheral recent thymic emigrants. Red dots and blue dots represent thymic$^-$ and thymic$^+$ patients, respectively. E) Linear regression analysis with TREC levels in blood against RTE % and F) CD31+ naïve CD4 T cell %. G) Linear regression with the proportion of double positive (DP) T cells in thymic tissue against RTE % and H) TREC copies in patient blood. I) Linear regression analysis of CD4 naïve T cells % and CD8 naïve T cell % against chronological age in < 50 years (young; green) (n = 35) and ≥ 50 years (old; purple) (n = 110) cohort. R^2 values calculated via univariate Pearsons's correlation coefficients and significance by two-sided p value analysis. J) RTE % in blood between smokers & non-smokers (left) and males & females (right) aged below 50. Truncated violin plots with quartiles, range and with significance evaluated by unpaired two-tailed t test.

Supplementary Figure 3. Inflammatory T cell profile in SAT. A) Gating strategy used to characterise inflammatory T cell populations in adipose tissue. Dead cells were excluded by LIVE/DEAD acqua staining, T cells were gated based on CD3 positive expression followed by CD4$^+$CD45RO$^+$ or CD8$^+$CD45RO$^+$ gating cell. TRM cells were defined bt CD69$^+$PD1$^+$ base on previous work(1). B) Cytokine producing T cell populations in SAT correlated to blood T cell populations and clinical characteristics. Significant correlations ($p < 0.05$) are shown as either red (positive correlation) or blue (negative correlation) dots. C-E) Association between CD4 IFNγ$^+$ T cell % in SAT and RTE%, CD4 TEM%, and CD4 TEMRA% in blood. F-H) Association

between CD8 IFNγ⁺ T cell % in SAT and RTE%, CD8 naïve%, and CD8 TEM% in blood. R^2 values were calculated using Pearson correlation coefficients and significance by two-sided p value analysis.

<u>Supplementary Figure 4.</u> Transcriptomic signature associated with thymic output. A-B) Principal component analysis (PCA) on gene microarray dataset showing no association between RTE High/Low phenotype and overall variation in global gene expression. C) Gene set variation analysis (GSVA) with RTE High and Low samples revealing differentially enriched gene signatures/pathways. D) GSEA pathway analysis between RTEs and MN T cells using Hallmark and E) GO biological process gene sets. Plots depict either most enriched, significant, or relevant gene sets. FDR-q < 0.25*.

<u>Supplementary Figure 5. Naïve T cell phenotype.</u> A) Representative histograms showing the expression of CD69 and PD-1 on the surface of $CD31^+$ and $CD31^-$ naïve CD4 T cells following stimulation with APC's and CytoStim™. B) Representative Dot plots showing CD69 and PD-1 co-expression in activated T cells. C-D) Representative Dot plots and histograms showing CFSE dilution on proliferating T cells. E) Representative histograms showing IFNy and TNFa upregulated in stimulated $CD31^+$ & $CD31^-$ naïve $CD4^+$ T cells and the proportion of $IFNy^+$ T cells co-cultured with or without IL-7. F) McPAS-TCR database search for matched alpha CDR3's to known antigens and their abundance compared to thymic output. R^2 values were calculated using Pearson correlation coefficients and significance by two-sided p value analysis.

<u>Supplementary Figure 6.</u> Association between epigenetic clock models and blood T cell populations. Significant correlations ($p < 0.05$) are shown as either red (positive correlation) or blue (negative correlation) dots. R^2 values for Pearson correlation are represented by dot size.

Reference

1.Vyas V, Sandhar B, Keane JM, Wood EG, Blythe H, Jones A, et al. Tissue-resident memory T cells in epicardial adipose tissue comprise transcriptionally distinct subsets that are modulated in atrial fibrillation. *Nat Cardiovasc Res.* 2024;3(9):1067-82.

Supplementary Figure 1

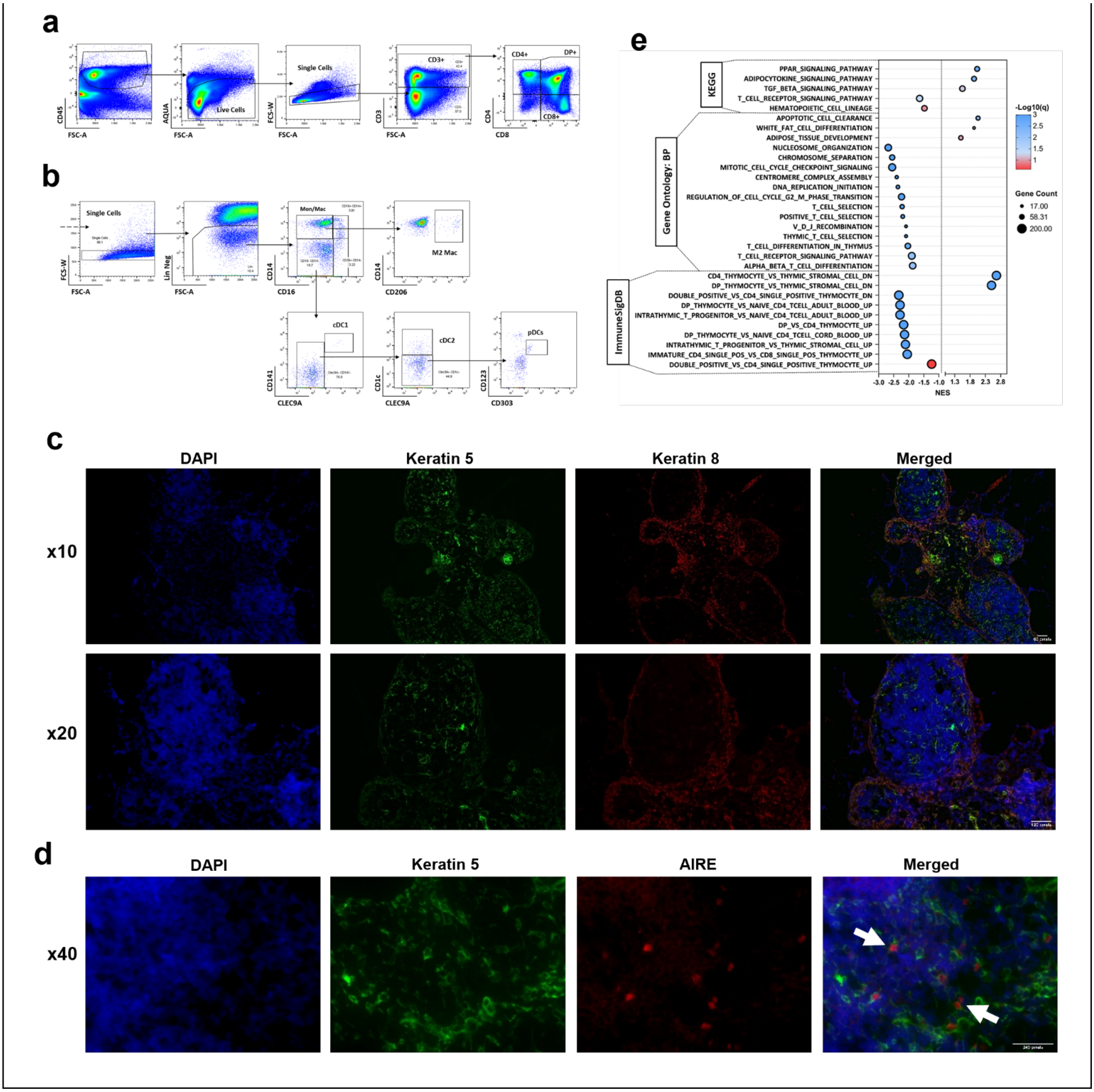

Supplementary Figure 2

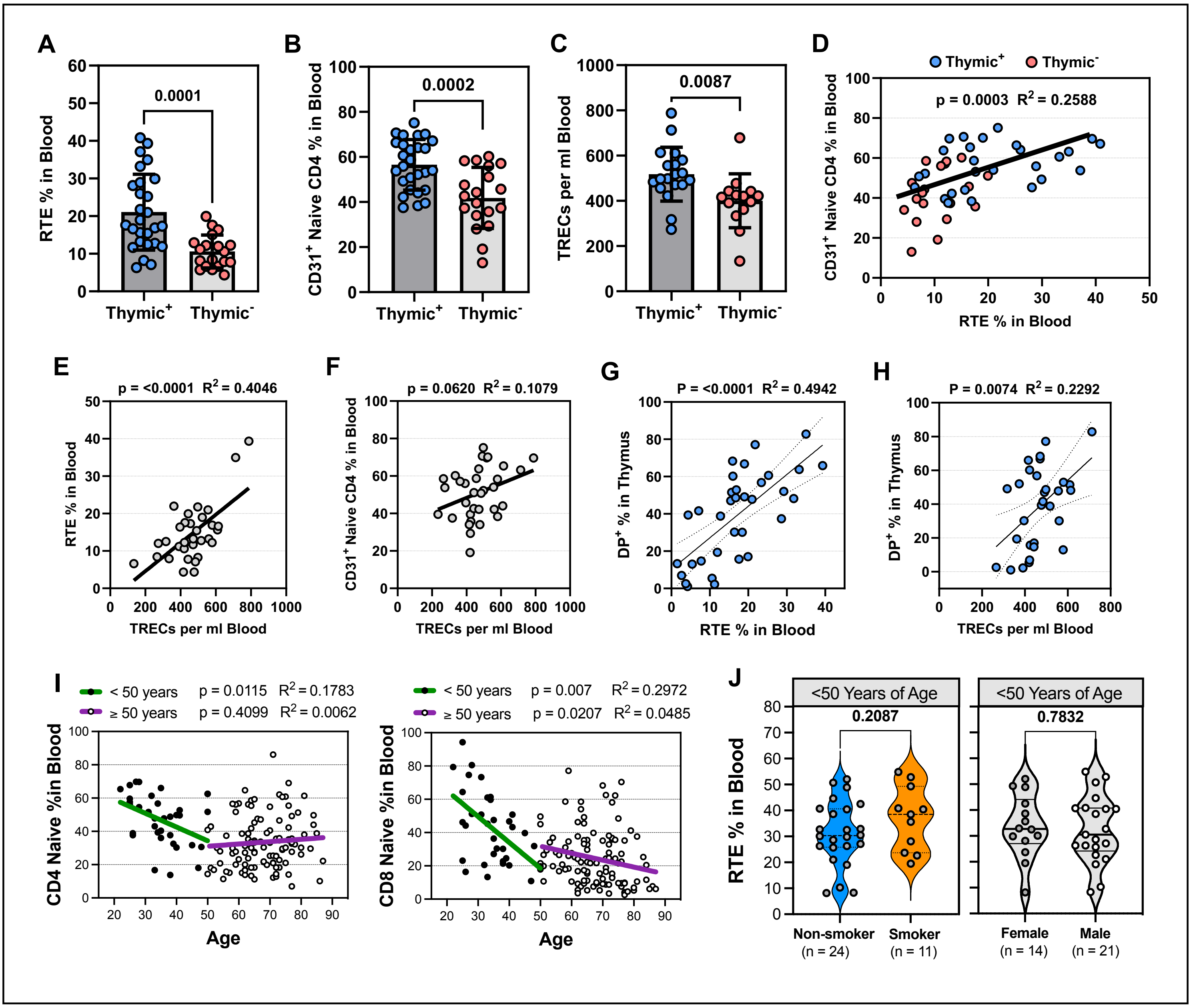

Supplementary Figure 3

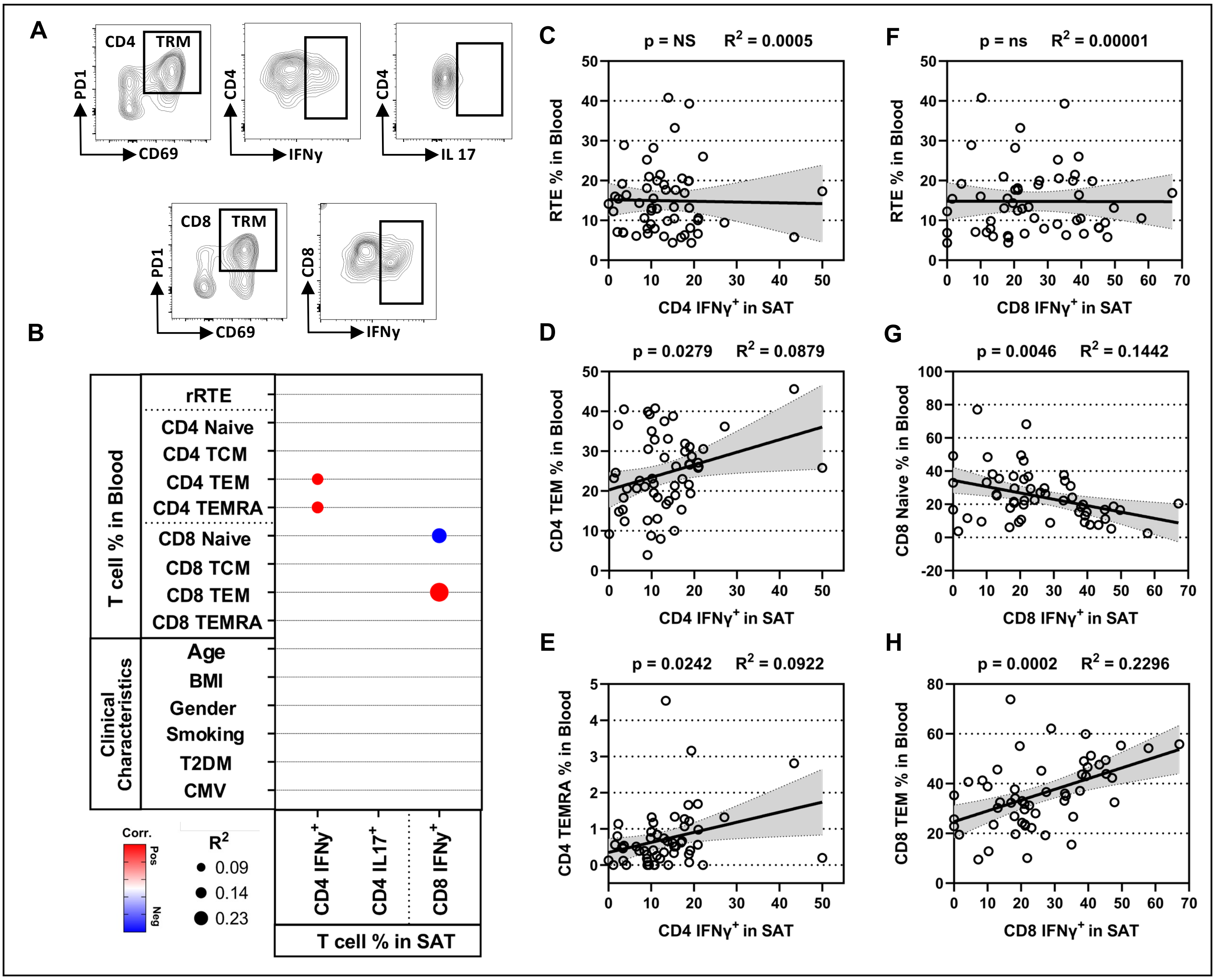

Supplementary Figure 4

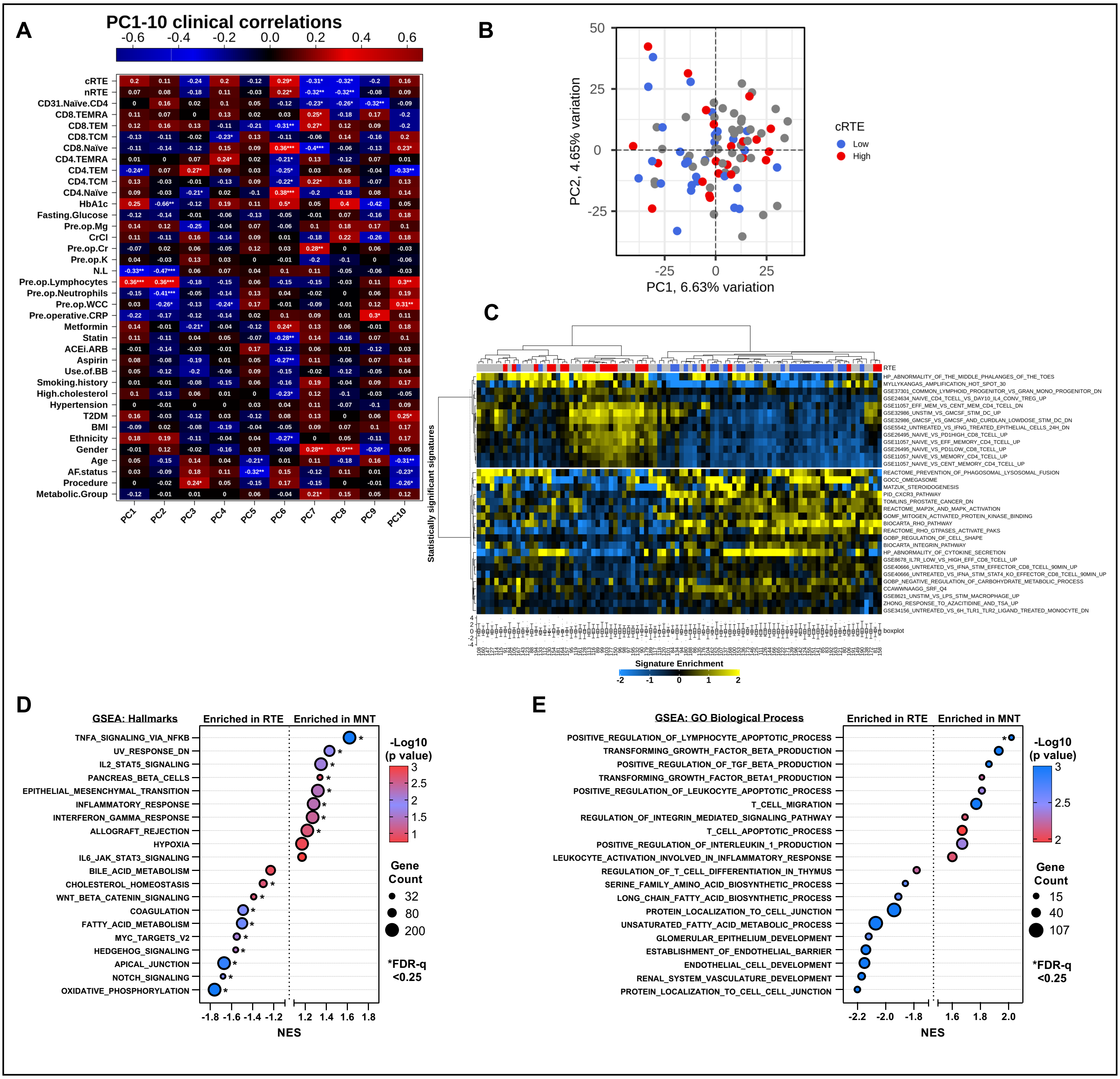

Supplementary Figure 5

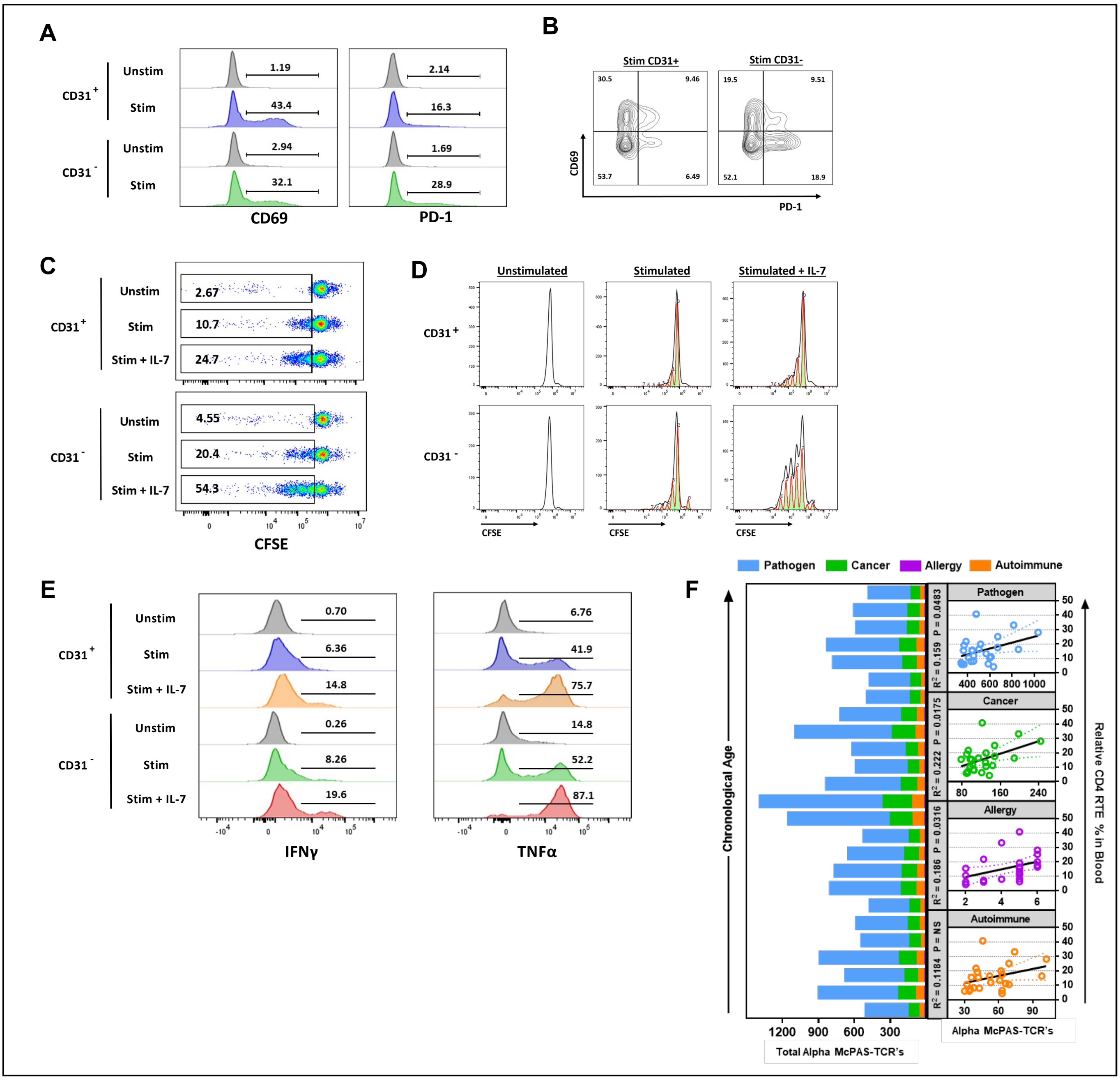

Supplementary Figure 6

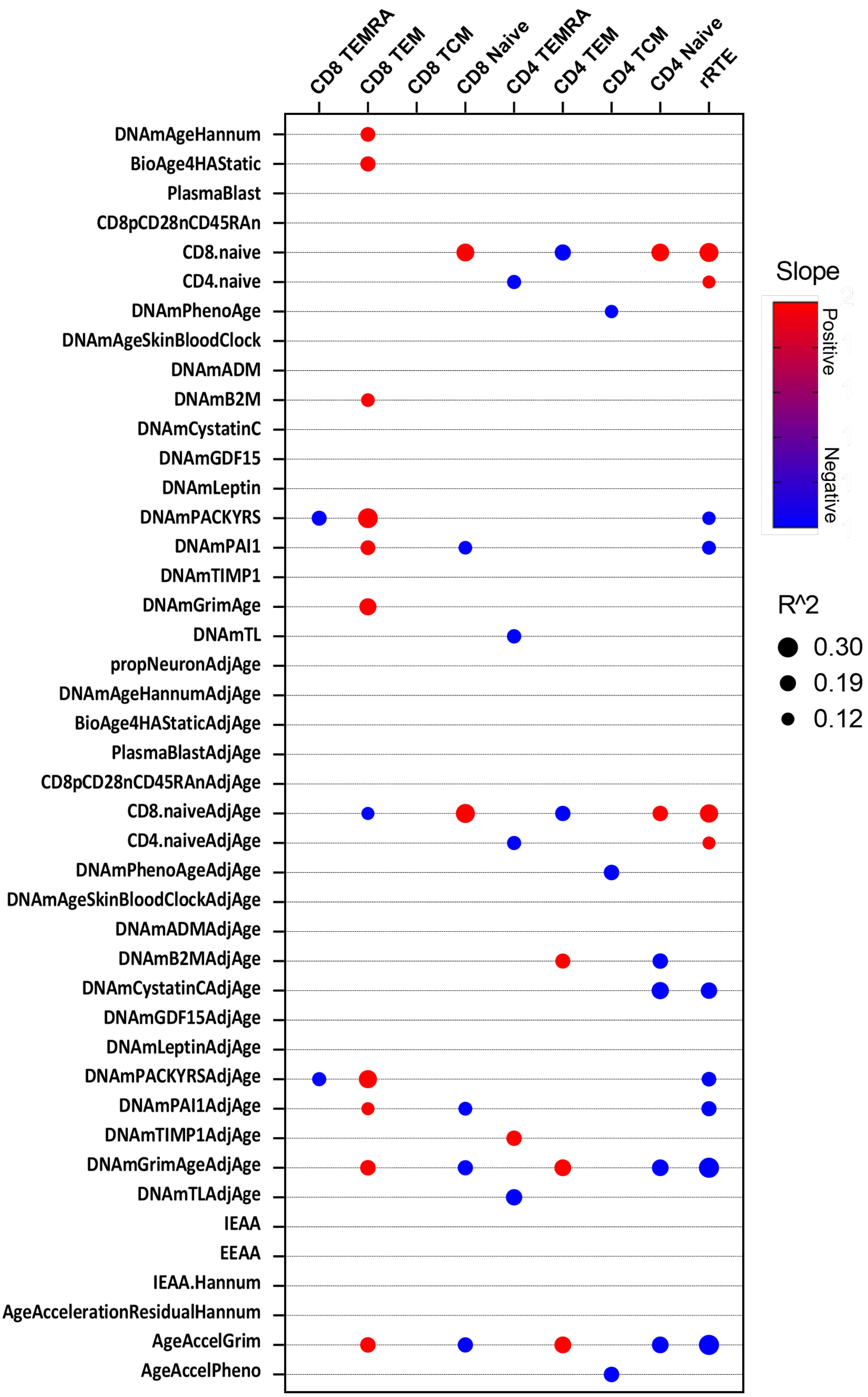

Supplemental Table 1

Clinical characteristics of patients characterised as either thymic+ or thymic- based on mediastinal adipose tissue phenotyping. Significance assessed via two-tailed t test (parametric), Mann-Whitney U test (non-parametric), and Fisher's exact test for categorical data. P<0.01**.

Clinical Variables	Thymic^{+} (n=27)	Thymic^{-} (n=19)	P Value
Age (Years)	70.96 ± 7.4	67.53 ± 8.5	0.1509
Male Gender (%)	13 (44)	17 (89)	**0.0022****
Body Mass Index (kg/m^2)	26.4 ± 5.4	28.7 ± 3.4	0.1045
Diabetes (%)	1 (3)	3 (15)	0.2916
Smoking History (%)	10 (37)	12 (63)	0.1336
Atrial Fibrillation Status (%)	17 (62)	10 (52)	0.5519
Hypertension (%)	21 (77)	13 (68)	0.5135
High Cholesterol (%)	20 (74)	12 (63)	0.5217
Pre-operative use of Beta Blockers (%)	16 (59)	10 (52)	0.7657
Pre-operative use of Statins (%)	21 (77)	13 (68)	0.5135
Pre-operative C-reactive protein (mg/L)	2.4 (0-12)	1.35 (0-5)	0.2108
Pre-operative neutrophil: lymphocyte	2.8 (1-5.6)	2.7 (1-6)	0.8844
GABG surgery procedure (%)	12 (44)	7 (37)	0.7628
Value surgery procedure (%)	15 (55)	8 (42)	0.5499
Combination GABG/VR (%)	0 (0)	4 (15)	n/a

Supplemental Table 3

Clinical characteristics of study patients

Clinical Variables	All Patients (n = 110)
Age (Years)	67.5 ± 9.2
Male Gender (%)	81 (73)
Body Mass Index (kg/m^2)	28.03 ± 4.7
Diabetes (%)	24 (22)
Smoking (%)	65 (59)
Atrial Fibrillation Status (%)	57 (51)
Hypertension (%)	79 (71)
High Cholesterol (%)	77 (70)
Pre-operative use of Beta Blockers (%)	69 (62)
Pre-operative use of Statins (%)	83 (75)
Pre-operative C-reactive protein (mg/L)	3.3 (0-12)
Pre-operative neutrophil: lymphocyte	2.9 (1-7)
GABG surgery procedure (%)	52 (47)
Value surgery procedure (%)	48 (44)
Combination GABG/VR (%)	10 (9)

Supplemental Table 4

Clinical characteristics of study patients

Clinical Variables	All Patients (n = 35)
Age (Years)	33.4 ± 6.2
Male Gender (%)	60 (21)
Body Mass Index (kg/m^2)	30.30 ± 9.4
Diabetes (%)	8.6 (3)
Smoking (%)	31.4 (11)
Atrial Fibrillation Status (%)	14.2 (5)
Hypertension (%)	14.2 (5)
High Cholesterol (%)	40 (14)
Left ventricular ejection fraction (%)	47.2 ± 18
Beta Blockers (%)	60 (21)
Statins (%)	31.4 (11)
Aspirin (%)	22.8 (8)
ACE inhibitors/ARBS (%)	42.8 (15)

Supplemental Table 5

Multivariate regression analysis of factors associated with %RTE

Variables	Odd ratio (95% CI)	*p* value
**Age**	1.008 (0.952-1.067)	0.7723
**Male Gender**	0.064 (0.012-0.338)	**0.0012****
**Body Mass Index**	1.022 (0.898-1.163)	0.7337
**Diabetes**	1.375 (0.373-5.055)	0.6316
**HbA1c**	1.218 (0.900-1.649)	0.1993
**Smoking**	0.240 (0.066-0.865)	**0.0292***
**Atrial Fibrillation (AF)**	3.150 (0.737-13.44)	0.1212
**Hypertension**	1.263 (0.369-4.315)	0.7093
**High Cholesterol**	1.015 (0.305-3.361)	0.9805
**Beta Blockers**	1.333 (0.388-4.575)	0.6478
**Statins**	0.888 (0.249-3.164)	0.8557
**Aspirin**	0.600 (0.194-1.854)	0.3750
**Statin**	0.888 (0.249-3.164)	0.8557
**ACEi /ARB**	0.934 (0.299-2.919)	0.9074
**C-reactive protein**	0.997 (0.902-1.107)	0.9957
**Neutrophil: Lymphocyte**	0.957 (0.655-1.399)	0.8239
**White cell count**	0.926 (0.707-1.213)	0.5782
**Lymphocyte count**	1.214 (0.528-2.790)	0.6474
**Neutrophil count**	0.854 (0.609-1.199)	0.3636
**Ischemic heart disease (CABG)**	0.981 (0.754-1.276)	0.9931
**Valvular heart disease (VR)**	2.407 (0.375-7.877)	0.1436
**Serum potassium**	1.014 (0.986-1.042)	0.3076
**Serum Magnesium**	0.376 (0.002-677.8)	0.7986
**Serum Creatinine**	1.001 (0.983-1.020)	0.8452

Supplemental Table 9

Multivariate regression analysis on the effect of %RTE in post-surgery recovery

Variables	Odd ratio (95% CI)	*p* value
**Day 1 WCC (10x10^9/L)**	0.985 (0.865-1.122)	0.985
**Day 2 WCC (10x10^9/L)**	1.135 (0.957-1.346)	0.145
**Day 3 WCC (10x10^9/L)**	1.039 (0.879-1.229)	0.651
**Day 4 WCC (10x10^9/L)**	0.933 (0.769-1.133)	0.370
**Day 5 WCC (10x10^9/L)**	0.698 (0.501-0.973)	**0.034***
**Day 1 CRP (mg/L)**	0.988 (0.968-1.008)	0.236
**Day 2 CRP (mg/L)**	0.990 (0.990-1.006)	0.574
**Day 3 CRP (mg/L)**	0.998 (0.992-1.004)	0.590
**Day 4 CRP (mg/L)**	0.997 (0.991-1.003)	0.370
**Day 5 CRP (mg/L)**	0.994 (0.983-1.004)	0.243
**LRTI**	0.245 (0.074-0.811)	**0.021***
**POAF**	1.350 (0.250-7.278)	0.7269

WCC, White Cell Count; CRP, C-Reactive Protein; LRT1, Low Respiratory Track Infection; POAF, Post-operative Atrial Fibrillation, CI, Confidence interval.
